# An Assessment of Pharmacy School Curricula in Florida and Inactivated Influenza Vaccine (IIV) Administration to Pregnant Women

**DOI:** 10.3390/pharmacy9010063

**Published:** 2021-03-18

**Authors:** Oluyemisi Falope, Cheryl Vamos, Ricardo Izurieta, Ellen Daley, Russell S. Kirby

**Affiliations:** 1 Merck & Co., Inc., Kenilworth, NJ 07033, USA; 2 College of Public Health, University of South Florida, Tampa, FL 33612, USA; cvamos@usf.edu (C.V.); ricardoi@usf.edu (R.I.); edaley@usf.edu (E.D.); kirbyr@usf.edu (R.S.K.)

**Keywords:** flu vaccination, pharmacy education, perinatal care

## Abstract

*Background:* There is a high risk for morbidity and mortality in pregnant women associated with influenza virus illness. Vaccine uptake rates in pregnant women remain lower than the targeted Healthy People 2020 goals despite recommendations from the Centers for Disease Control (CDC). Few studies have examined the role of the pharmacist in providing immunization services to pregnant women, fewer still have directly examined the PharmD curricula and the perspectives of pharmacy students on how they perceive their role in providing influenza inactivated vaccine (IIV) to pregnant women. *Objective:* This study examined the PharmD curricula instruction with regard to immunizing pregnant women and how pharmacy students perceive it. *Methods:* Semi-structured, in-depth, in-person qualitative interviews were conducted with the six Academic Deans of the accredited schools of pharmacy in Florida, and three focus group sessions were held with third- and fourth-year pharmacy students (*n* = 18) in Florida. A thematic analysis was conducted. *Results*: Most academic deans reported providing instruction on immunization in schools with respect to vaccine administration in pregnant women and called for a need for all schools to make it compulsory to include pregnant-women-specific content. Pharmacy students reported a gap in knowledge of content related to administering the IIV in pregnant women, but feel that when presented with the opportunity, they will be willing to provide IIV to pregnant women. *Conclusions*: Pharmacists are in a good position to play a role in increasing IIV rates among pregnant women. Implications for practice include the need for incorporation of pregnancy-specific content to immunization curricula.

## 1. Introduction

A key epidemiologic finding during influenza epidemics and pandemics is an increased risk for severe morbidity and mortality in pregnant women and their fetuses [1,2]. Influenza infection in pregnant women lead to complications such as pneumonia, spontaneous abortions, preterm births, birth defects, and fetal loss [3,4]. The Center for Disease Control (CDC) recommends Inactivated Influenza Vaccine (IIV) for pregnant women [5,6,7]. Despite the recommendation, vaccine uptake remains suboptimal, about 50%, and is lower than the targeted 90% coverage proposed by the Healthy People 2020 goal [8,9,10,11]. Uptake of IIV during pregnancy also protects the baby for its first few months of life, as there is no influenza vaccine for infants under six months of age [12].

Interventions such as reminders have been employed to improve influenza vaccine uptake in pregnant women [13]. However, barriers including access, distance to care centers, no transportation, and non-availability of the vaccine at care providers clinic still pose an issue [14,15,16,17]. Pharmacists can provide immunization services through the standing orders program, which is a recommendation of the CDC’s Advisory Committee on Immunization Practices, which allows other healthcare providers besides physicians to administer vaccines according to a physician/institutional approved protocol [18]. The Accreditation Council for Pharmacy Education (ACPE) requires specific topics be covered in required content areas for a school to be accredited, which include immunization under public health [19,20]. Most pharmacy schools now provide immunization training [21], and instruction is delivered through the American Pharmacists Association (APhA), and programs such as the Minnesota College of Pharmacy Immunization Delivery Program, and the Collaborative Education Institute Immunization Training [20,21]. Studies show that pharmacy students can successfully administer vaccines to adults when trained [22]. However, when it comes to pregnant women, studies have demonstrated reluctance by pharmacists [23].

This study aimed to explore the course content and perception of pharmacy students regarding pharmacy school curricular training on vaccine administration to pregnant women with a focus on the IIV. This was carried out through exploring the curricular and perceptions of pharmacy students on administration of IIV to pregnant women using the Theory of Planned Behavior (TPB) as a guiding framework. The TPB is used to demonstrate why an individual would or would not engage in a behavior [24]. The determinants of intention to engage in a behavior, in this case administering IIV to pregnant women (constructs of the TPB), are behavioral attitudes (beliefs regarding providing IIV to pregnant women and the notion that doing so has specific outcomes), social norms (notions of how certain individuals view and approve or disapprove of pharmacists providing immunization services to pregnant women), and the perceived behavioral control (how much the students believe providing IIV or not providing IIV to pregnant women is under their volitional control), regarding the behavior [24]. For this study, the TPB framework is expanded to include “knowledge” as one of the constructs to be tested, because subjective norms have been considered to be a weak predictor of intention [25].

## 2. Methods

### 2.1. Participants

This qualitative research study employed interviews with academic deans and focus groups with students. Drawing from the TPB, the focus group guide asked questions about knowledge, attitudes, subjective norms, and perceived control regarding administering IIV to pregnant women. Academic deans and pharmacy students in the six accredited schools of pharmacy in the state of Florida were chosen as the target population. The academic deans were chosen as they are usually responsible for the school curricula and are the most knowledgeable about it. For the pharmacy students, the sampling frame included third- and fourth-year pharmacy students at the accredited schools of pharmacy in Florida, as they are believed to have completed the majority of their coursework. They were recruited via purposive sampling. All 6 academic deans participated, and for the focus groups, saturation was reached after 3 focus groups with 18 participants. The study was approved by the University of South Florida Institutional Review Board (IRB).

### 2.2. Data Analysis

Interviews were conducted by OF, and focus groups were also facilitated by OF and a research assistant, who took also notes and helped coordinate the sessions. The interview and focus group guides were designed to be open-ended, to allow participants describe their personal opinions, perceptions, and experiences regarding inactivated influenza vaccine administration in pregnant women knowledge and practices. Demographic information was collected from the students, and nomenclature of courses that provided immunization content were also collected from academic deans. Recorded interviews and focus groups were transcribed. A distinctive identifying number was used to differentiate participants. MAXQDA software was used for data management and analysis. An a-priori code book was developed that had themes based on content and the TPB. Analysis began as interviews were being conducted and transcribed, and emergent codes were added to the code book with revised definitions as needed, and then finalized. Analysis was carried out by two independent coders to produce an inter-rater reliability measure of 84%.

## 3. Results

### 3.1. Review of Pharmacy School Curricula (Academic Deans)

#### 3.1.1. Course Content on Immunization

All participants reported they offered required courses on immunization and a couple (*n* = 2.33%) reported offering elective courses with additional information on immunization. Names of courses were provided (Table 1). Courses included online and in-class modules, course readings, trainings, and videos on immunization.

All respondents utilize APhA modules to complement immunization training. Five (83%) participants reported that immunization teaching and certification were offered in the first year, while 17% (*n* = 1) reported it was offered in the second year.

“We also have the students do a certification course through the APhA and through that course the students are certified to give immunization … and after finishing the self-course, they would demonstrate their technique, they would get assessed on it, a faculty member will do the assessment and the evaluation and all of that is sent to APhA, and APhA will give the certification to the student.” AD2.

When asked if their pharmacy schools offered immunization classes specific to pregnant women, four respondents (67%) mentioned that immunization content specific to pregnant women are covered under special populations, which include pregnant women, however two mentioned that immunization content is not necessarily focused on pregnant women.

“Not necessarily a focus on pregnant women, the gist of it is about the administration technique, and the different types of vaccines that pharmacists can actually administer, and then there is a special populations discussion, but it is not, you know, like a situation where there is any push to increase administration in pregnant women or anything of that nature.” AD1.

#### 3.1.2. Compulsory Immunization Teaching

Respondents reported that all schools should now be teaching immunization as part of their curriculum in accordance with the ACPE accreditation standards. When asked about making it compulsory for the School of Pharmacy curricula in the U.S. to include content material on immunization specific to pregnant women, all respondents (100%) reported pregnancy-specific information should be made compulsory. One respondent reported it would be a way for pharmacy students to become advocates for vaccine uptake in this population.

“One of the suggestions for pregnant women is always to get flu shots, our students, a lot of them do rotations in women’s health, also they do rotations in the hospital, even in the community just as an advocate for preventive health and we stress that people get their flu shots, so, it is very important they become advocates and do that.” AD5

#### 3.1.3. Sufficiency of Immunization Training and Role of Pharmacists

Regarding the training on immunization being enough to equip students to get certified, all participants (100%) agreed the coursework together with the APhA modules are sufficient to equip and prepare the students to be certified for immunization. One participant mentioned it will also depend on their experience certification to get them more comfortable with administering vaccines, and their subsequent employers to get them more training.

“tT get the certificate they have to do a demonstration of actually administering the vaccine, and then that allows them to go to their training sites to participate, but once they get licensed, then they just become responsible for maintaining the certification, and they have to just get more CEs (Continuing Education).” AD1.

When asked about perceptions regarding pharmacists administering influenza vaccines to pregnant women, all participants felt that it was something pharmacists should do. Three (50%) mentioned pharmacists being accessible and therefore it would be easier for pregnant women.

“I believe they are very much qualified regardless of whether the patient is pregnant or not. And it is something they can do and should do.” AD4.

### 3.2. Pharmacy Student Focus Groups

#### 3.2.1. Demographics

For the demographics, 18 participants from three of the six accredited schools of pharmacy in Florida participated in the focus groups (Table 2), 83.3% (*n* = 15) of them were female and 16.7% (*n* = 3) were males. All participants had received instruction on immunization, 83.3% (*n* = 15) mentioned they had received instruction regarding immunization in pregnant women, while 16.7% (*n* = 3) were not sure if they had. All but one participant was certified to immunize, and none had provided vaccination to a pregnant woman.

#### 3.2.2. TPB Constructs and Influenza Vaccine Administration in Pregnant Women Practices

Knowledge: Students described knowledge of the mechanism of action, types of the vaccine, who should get it, and misconceptions surrounding the vaccine (Table 3). Regarding what the students knew about the influenza vaccines and pregnant women, they all agreed the vaccine was safe in pregnant women, as long as it was the inactivated vaccine. A few participants were not sure pregnancy-specific content was taught; students expressed the need for pregnancy specific training in their curricular (Table 3).

Attitudes: Students described positive and negative attitudes. Participants believed they are in a position to provide immunization services effectively, not just in terms of accessibility of pharmacists and availability of the vaccine, but to provide expert advice Table 3). Another theme that came up was the ease for the patients. Participants expressed concerns such as they may have limited information about pregnant women and fear of liability (Table 3).

Perceived Control: Participants were asked about the facilitators or barriers to providing the influenza vaccine to pregnant women. Healthcare provider recommendation was perceived as a facilitator across all focus groups. Other facilitators that were mentioned include the use of marketing/incentives (Table 3). When asked about barriers, students expressed various sentiments including lack of a relationship or trust between pregnant women and the pharmacist, lack of awareness by pregnant women of the safety of the vaccine during pregnancy, lack of time, and lack of education of themselves as pharmacists regarding providing vaccines to pregnant women (Table 3). Other themes include the type of insurance or the community settings (rural, urban) of the pregnant woman. When asked about the patient’s location or community settings (rural vs. urban), students across all three focus groups believed that in rural areas patients may not be well informed, have transportation, or be able to afford the vaccines (Table 3).

Subjective Norms: The students were asked about the influences of the following groups on them providing influenza vaccines: Pregnant women the CDC, APhA, Florida state statutes, physicians, pregnant women, and their peers. Regarding the CDC, students believed they played a role as a resource for information to keep up with guidelines and changes regarding the influenza vaccine administration. About APhA, students believed they are a key resource through which they obtained their training on immunization and for continuing education, and also expressed the need for the APhA training to focus more on special populations (Table 3). When asked about the influence of the Florida state law regarding pharmacists providing immunization services under the supervision of a Physician within a framework of an established protocol [26], students expressed mixed feelings on how it affects their vaccine administration practices, as they feel it takes away from their autonomy and may not be necessary. One participant expressed that the arrangement is useful for the pharmacist in terms of liability (Table 3). When asked about the influence of the physician, students believed if physicians refer pregnant women, they will feel safer coming to the pharmacy for their shots, and some believe doctors are already referring patients to go to the pharmacy to receive the influenza vaccine and others expressed the need for communication between pharmacists and doctors (Table 3).

Regarding the influence of pregnant women, none of the participants had prior experience providing immunization in pregnant women but expressed that pregnant women may not be comfortable getting their immunizations from the pharmacist. Others felt that with proper counseling pregnant women will be comfortable getting their shots from a pharmacist. Regarding peer influence, students believed that their colleagues choosing not to provide immunization services to pregnant women may not affect them personally, but they expressed that when pharmacists do not immunize, it holds the practice of pharmacy back and pharmacists who do not immunize pregnant women tend to be older and set in their ways. They also expressed that when pharmacists immunize it would increase the number of women receiving the vaccine and will reduce missed opportunities (Table 3). Regarding projected future practices, when asked about if they would be willing to administer influenza vaccines to pregnant women, all participants expressed a willingness to administer influenza vaccines to pregnant women and most believed it would not feel different from giving a non-pregnant person the vaccine but may administer with more caution (Table 3).

## 4. Discussion

Connecting the Academic Dean’s interview findings with the students’ focus groups allowed for a better understanding of pharmacy school curriculum in Florida. The focus groups provided an insight on how the curriculum is translated to the pharmacy students who received the instruction. The qualitative methods employed in this study best address the issue of cognitive understanding or perceptions of course instruction on immunization in their pharmacy school courses.

The pharmacy school’s curriculum review findings showed that all six schools provide instruction on immunization as part of the ACPE requirements, however 67% percent of the schools provided immunization content with respect to pregnant women in their required courses. All academic deans reported that pharmacy schools should include compulsory immunization content with a focus on vaccines in pregnant women. Students also reported they would like to receive more information about influenza vaccine administration to pregnant women, suggesting immunization content may lack or not have focused pregnancy content.

In discussing students’ attitudes about administering IIV to pregnant women, they believe they are accessible and qualified to provide such services and receiving immunization from pharmacists may be more cost-effective for pregnant women. This is consistent with previous research by Steyer et al. (2004), which shows pharmacists are the most accessible health care provider [27]. Other studies have demonstrated the cost effectiveness of pharmacists providing immunization [28,29]. Students also expressed concerns such as not having enough information about the women, fear of liability, and as students they feel they might be limited in their knowledge to provide influenza vaccine to pregnant women. Dolan et al. (2012), also reported that pharmacists reported fear of liability when it came to providing immunization to pregnant women [23].

For the perceived control, students reported facilitating factors to include provider recommendation and campaigns/incentives. High rates of influenza vaccine uptake among pregnant women are associated with provider recommendation [30]. Pharmacists advocating or campaigning for vaccines save costs and prevent hospitalization [28]. In discussing barriers, students mentioned pregnant women may not trust the pharmacist. This contrasts a study that showed pharmacists being one of the most trustworthy healthcare providers [31]. Other barriers include time constraint, not enough knowledge about immunizing pregnant women, insurance type, pregnant women being hesitant, and their community settings. A study also reported that pharmacists believe pregnant women may not be interested in the vaccine and the patient’s insurance may not cover it [23]. Another study reported accessibility of pharmacists administered influenza vaccines to individuals residing in rural areas [32], which was not a sentiment brought up during the focus group discussions.

In discussing the subjective norms, the CDC and APhA were reported to provide guidelines and training respectively and enable students provide influenza vaccines to pregnant women. This resonates with a study that reported pharmacists were willing to provide immunization to pregnant women because it complies with the CDC guidelines [23]. However, students expressed the need for training to focus more on pregnant women. Students expressed that pregnant women may prefer to get vaccines from their doctors. When discussing the Florida state statutes influence, students had mixed feelings on how it affects their vaccine administration in pregnant women practices but appreciate relevance of it. This resonates with studies that reported that pharmacists providing immunization is facilitated by the standing order [27,33,34].

Overall, Academic Deans and pharmacy students shared the sentiments that they are equipped to provide immunization services. Despite the call for the need for inclusion of pregnancy-focused immunization training, students believe that when presented with the opportunity, they will be willing to provide influenza vaccines to pregnant women. This resonates with studies that assessed pharmacy students and showed they gained self-confidence in administering vaccines and can boost vaccine uptake rates [22,35].

## 5. Conclusions

The study had limitations. Firstly, no two students remember or learn the same way, therefore perception on instruction may be subjective. In addition, there may have been recall bias for the students, as immunization instruction was received in year 1 or 2 of their program. The qualitative nature of this study is important in examining how students perceive what they learn and how they may relate it to their future practice. In addition, this study provided an opportunity not to only examine students’ perceptions, but also the perceptions of the Academic Deans of pharmacy schools in Florida. In addition, the study was guided by the theory of planned behavior. The philosophy of this framework as applied to pharmacists providing influenza vaccines to pregnant women, is that a pharmacists’ ability for influenza vaccine administration to pregnant women may vary based on the volitional control, normative beliefs, preconceived notions, perceived barrier, or facilitator of offering and administering the vaccine to pregnant women. Based on this philosophy, the constructs of the TPB framework include pharmacy students having the knowledge about the influenza vaccine in pregnant women; having the right attitudes about providing the vaccine to pregnant women; understanding and being able to navigate the barriers to providing these services; and the subjective norms that support their role to provide these services are the best predictors of pharmacists providing immunization to pregnant women. This suggests that these constructs are applicable to studying pharmacists and other providers and their intentions to provide a healthcare service, and also have important implications for both interventions and prevention efforts with at risk groups, such as pregnant women. This is a positive finding in light of the knowledge that pharmacy schools cover immunization in their curriculum, and some more than others provide content specific to pregnant women; also, students believe they can play a role in providing influenza vaccines to pregnant women. However, in this study, few schools do not provide immunization content specific to pregnant women, and students demonstrated knowledge gaps, which suggest there is still more work to be done.

Furthermore, this study included the perception of individuals i.e., pharmacists that are often overlooked when it comes to providing care for pregnant women. Pharmacists are in a good position and play an important role in tackling the issue of low influenza vaccine uptake among pregnant women rates. Since pharmacists already provide immunization to adults, administering vaccines to pregnant women should not be an issue. Pharmacists undergo the required training and obtain the required skills to provide such services. It has also been demonstrated that utilization of immunization services by pharmacists are cost effective and provide accessibility, therefore pharmacy schools and the ACPE should review opportunities and strategies to enhance vaccine training for special populations including pregnant women.

## Figures and Tables

**Table 1:** A typology of courses that contain content on immunization.

Participant	Required Courses	Year Offered	Pregnancy Content
AD1	Foundations of Public Health	PG Y1	NA
AD2	Immunology and Infectious Disease	PG Y2	
	Population Health	PG Y1	Covered
AD3	Applied Patient Care	PG Y1	
	Pharmacotherapeutics III	PG Y3	Covered
AD4	Integrated Pharmacy Application I	PG Y1	
	Integrated Disease Management	PGY2	Covered
AD5	Public health medication safety and disease prevention	PG Y1	Covered
AD6	Professional Practice Skills lab	PG Y1	NA
	Endocrine women and men’s health	PG Y2	NA
Participants	Elective Courses	Year Offered	Pregnancy Content
AD3	Vaccine and Immunizations	PG Y2	Covered
AD6	Women’s Health	PG Y2	Covered

PG (Post Graduate), Y (Year).

**Table 2:** Pharmacy student focus group demographic data.

Variable	*n*	Percent
What is your age range?		
18–30	15	83.3%
31–40	3	16.7%
What gender do you identify as?		
Male	3	16.7%
Female	15	83.3%
Did you take any classes in your Pharm D program which taught you about immunization?		
Yes	18	100%
No	0	0%
Have your pharmacy school classes discussed immunization in pregnant women?		
Yes	15	83.3%
No	0	0%
Not sure	3	16.7%
Are you certified to administer vaccines?		
Yes	17	94.4%
No	1	5.6%
Have you ever administered a vaccine before?		
Yes	9	50%
No	9	50%
Have you ever administered a vaccine to a pregnant woman before?		
Yes	0	0%
No	18	100%

**Table 3:** Theory of Planned Behavior (TPB) major themes and subthemes of pharmacy students focus group findings and representative quotes.

Themes	Representative Quote
**Knowledge** Mechanism of action	“… the body is able to mount an immune response to the inactivated or the live attenuated vaccine and by mounting that response you then gain immunity to the strains within that vaccine through the formation of antibodies, so that if you are exposed to it again the body is able to defend against it …”
Types of vaccine	“… there is the live vaccine and there is the inactivated vaccine. Depending on what the patient’s position is, you may not want to give one over the other and there are like different formulations that are better for the younger population and some that are better for the older. There is the trivalent, quadrivalent and all that, they come in different strengths and different amounts.” “We have the data and the science that it is safe and effective, and it is not going to be harmful as long as it is an inactivated vaccine …”
Recommended groups	“Everybody should get it. When they are of age … Well I mean you are not going to give it to like a newborn …”
Misconceptions	“It takes a couple of weeks to actually have a full effect of the immunization, so if you get sick during those two weeks after having the flu shot, it is not because of the vaccine, but because of the flu virus itself, and you just happen to get it during that time. It is a common misconception …”
Instruction on pregnancy vaccine	“if they included more patient specific populations in immunization training, pharmacists may feel more comfortable …”
**Attitudes**	
Access	“… pharmacists are easily accessible in the community settings it is a huge advantage for patients to be able to gain access to receiving immunizations. It really opens up as far as the number of immunizations we are able to provide …”
Experts	“I think we are qualified just in the knowledge that we are providers … Also, we can touch up on things that fall under our scope such as vaccines”
Ease for patients	“It’s less cost if you think about it because they have to make an appointment with their doctor which they have to do a co-pay and then, like, get the vaccine and … it just, it creates less cost for the patient.”
Limited information	“… we don’t know the background of the patient as maybe we should”
Limited knowledge	“… We can talk about like Flu shot, as far as our knowledge at least now as students we are kind of like limited.”
Liability	“I think there could possibly be more liability associated with administering vaccines to pregnant women because of adverse effects whether it is related to the vaccine or otherwise. It could be definitely a liability issue and it could come back to the pharmacist regardless of if they administered it appropriately for that patient. It is just higher risk for the pharmacist’s license”
**Perceived Control**	
Facilitators Provider recommendation	“If they received a note from the doctor, and they speak to their doctor about why it is okay to receive the vaccines from pharmacists and why they should do it, that may make them more likely to”
Marketing	“… A poster like ‘hey, you can get it here, but you can also go to your Pharmacist and get it there’.”
Barriers	
No trust	“… It is a trusting relationship, like they have that relationship with the doctor. So, they will probably feel safer getting it there.”
No awareness of vaccine safety	“One of the barriers is patients lack information, they may say I am pregnant why would I get a vaccine that’s going to hurt the baby”
No time	“the pharmacists feel pressured to meet that number and if they don’t have a pharmacy intern or anybody else to give those vaccines, they have to constantly be leaving the workflow in order to administer vaccine …, it is a pretty time-consuming process.”
No education	“I guess where I am at right now, probably lack of education, we never really focused much on the pregnant women, at least in our education so far …”
Community settings	“… they (rural dwellers) might also, maybe not have enough access to information regarding vaccination, either through their provider of through basically any other resources they could obtain in a more urban setting.”
Insurance	“Insurance doesn’t always cover it. We have to put in particular codes on the computer and sometimes, depending on the vaccine, and depending on the insurer/insurance coverage, Insurance won’t always cover it. They won’t cover like a Pharmacist giving it.”
**Subjective Norms**	
CDC	“We go to the CDC for updated guidelines for what has changed regarding the vaccine or regarding a special population. I feel like they are the most updated information” “I am not sure as far as re-certification goes, but as far as our previous certification and initial training in APhA, they could focus more on special population”
APhA	“I am not sure as far as re-certification goes, but as far as our previous certification and initial training in APhA …, they could focus more on special populations …”
Florida State	“… I guess it does kind of put the provider at some risk for liability, but in my experience, I have always thought it was very convenient that we have that, and it is very helpful to the pharmacist” “We are a long way from Pharmacists being considered practitioners. We are not even considered practitioners. So, for us to be able to vaccinate, we had to put something in place.” “I don’t know … I feel like it’s not necessarily like a “bad thing” like in case of I guess in case of “special cases” or whatever. Then again, it also kind of hinders …”
Physicians	“Yeah, coming from them (physicians), they (pregnant women) feel more safe doing it at the pharmacy.” “I think as far as we are in communication with each other, it will take some load of the OBGYNs and if they pharmacists have questions to be able to contact whenever, and say ‘hey, we are giving this patient this vaccine is that okay with you, and then they proceed in that way’. And keeping contact with the patient all in the same loop.”
Pregnant Women	“… The chances are they are probably getting it from their doctor, because I feel like the woman is going to be more in touch with their OBGYN.” “I personally wouldn’t know how to approach a pregnant woman who had concerns, I would definitely grab my boss who is a pharmacist and kind of “tag-team”—as I call it—and counsel her and hopefully, with what we explain to her she’d be comfortable getting immunized.”
Peers	“I know that the older generation of Pharmacists weren’t really too keen-at least some of the pharmacists that I’ve worked with who’ve been on the field for twenty plus years … “so, I have to give vaccines now?” It’s not something that I think they care to do … But the newer generation of Pharmacists are definitely more on-board for doing something like that.” “I think that pushes us back, because I actually had a pharmacist who told me that she refused to give vaccines when it was first started for like a solid two years. Like patients would come in to ask for the vaccine and she would turn them away. I feel like it makes the patient, probably feel some type of way that a pharmacist with a doctorate is refusing to give the vaccine, so, are all pharmacists capable of giving this vaccine? or what is the reason behind it, I do think that does push us back. And I feel like those pharmacists are set in their ways and they really don’t want to expand our scope of practice”
**Projected Practice**	Participant 1: “I would be fine with it.” Participant 2: “Yeah.” Participant3: “I would be fine with it too … I personally would probably want to do a little bit more research to make sure that the vaccine that I am giving is safe.” Participant 4: “But I think we have access to the appropriate resources so that wouldn’t be a problem.” Participant 5: “Yeah, I would definitely have to consult that resource before I do anything but then as long as it says it is fine, I would have no problem doing it. Participant 6: “I would be comfortable. I personally would probably double and triple check myself as far as the vaccine is safe to give.” Participant 7: “Yeah, as long as there is not any other co-morbidity or anything wrong with that woman’s pregnancy … and same with another patient that might have another co-morbidity—I would want to make sure that everything checks out before giving the vaccine.” Participant 8: “… but giving it to a pregnant woman, I would just treat them like any other patient.” Participant 9: “Yeah, pregnant or non-pregnant person, it is the same thing”. Participant 10: “I will feel fine as long as I double check and the pharmacist manager says it is alright, so it will be like just administering the vaccine to any other patient.”

## Data Availability

All dataset analyzed, and records were stored on the University of South Florida Box folder. Data available on request due to privacy restrictions. The data presented in this study are available on request from the corresponding author. The data are not publicly available due to IRB privacy restrictions.
